# Early diagnosis of spinal tuberculosis by magnetic resonance: perfusion weighted imaging in a rabbit model

**DOI:** 10.1186/s12880-022-00870-x

**Published:** 2022-08-09

**Authors:** Xiaochen Liu, Yunlin Wang, Wenxiao Jia

**Affiliations:** Imaging Center, The First Affiliated Hospital of Xinjiang Medical University, No. 137, South Liyushan Road, Xinshi District, Urumqi, 830000 Xinjiang China

**Keywords:** Spinal tuberculosis, Perfusion weighted imaging, Perfusion parameters, Hemodynamics

## Abstract

**Background:**

This study aimed to analyze the application value of magnetic resonance (MR)-perfusion weighted imaging (PWI) in the early imaging diagnosis of rabbit spinal tuberculosis.

**Methods:**

Spinal tuberculosis model was established using ATCC25177 *Mycobacterium tuberculosis* strain in the lumbar spine of rabbits. Forty rabbits were divided into 2 groups: rabbits in the experiment group were injected with 0.2 ml of 5.0 mg/ml tuberculosis suspension (n = 30) and those in the control group were injected with 0.2 ml of normal saline (n = 10) after vertebrae drilling surgery. Routine MRI and MR-PWI were performed at 4, 6, and 8 weeks after surgery. The statistical difference in terms of perfusion parameter values in the early MR-PWI scan of spinal tuberculosis between two groups was analyzed. The receiver operating characteristic (ROC) curve analysis was conducted for the accuracy of MR-PWI parameters in the early diagnosis of spinal tuberculosis.

**Results:**

Except time to peak, the other perfusion parameters in the experiment group were all increased with time. In addition, the difference between the two groups, as well as the differences at each time point was statistically significant (all *P* < 0.05). First-pass enhancement rate (Efirst), early enhancement rate (Ee), peak height (PH), maximum slope of increase (MSI), maximum signal enhancement rate (Emax) and signal enhancement rate (SER) showed high values in early diagnosing spinal tuberculosis.

**Conclusion:**

The parameters including Efirst, Ee, PH, MSI, Emax and SER may provide valuable imaging evidence for the early diagnosis of spinal tuberculosis in clinical application.

## Highlights

1. A rabbit spinal tuberculosis model was established.
2. Various parameters in the early MR-PWI analysis were different in two groups.
3. MR-PWI may provide valuable imaging evidence for diagnosis and treatment of spinal tuberculosis.

## Background

Spinal tuberculosis as a destructive form of tuberculosis accounts for 15% of extra-pulmonary tuberculosis [1]. The incidence of it in many parts of the developing world remains high and is increasing in developed countries due to migration and emergence of multidrug resistance strains [2]. Since the slow and insidious disease progresses of spinal tuberculosis, the diagnosis usually takes weeks or even years. Rapid and effective diagnostic method is particularly important.

The manifestation of spinal tuberculosis in the early stage is diffuse inflammatory edema, exudation, proliferation and caseous necrosis [3], followed by vertebral bone destruction, narrowing of intervertebral disk space and paraspinal abscess [4]. Currently, there are several methods for diagnosing spinal tuberculosis, but they all have some disadvantages. The histopathology and genetic tests have low sensitivity, and the diagnoses based on clinical symptoms and laboratory tests have low specificity. In addition, although the histopathological method has been applied to research the blood supply status of the diseased area and trauma [5, 6], the results obtained can only reflect the angiogenesis in a very small area, and could not dynamically evaluate the angiogenesis activity of the diseased area. MRI scan is often applied in blood perfusion examination in tissues or organs [7, 8]. Perfusion weighted imaging (PWI) which could provide hemodynamic information that cannot be obtained by conventional MRI and magnetic resonance angiography is often used to study the distribution of microvessels and capillary blood perfusion [9, 10]. Currently, MR-PWI has been widely used in clinical application, including brain tumors, myocardial ischemia, nasopharyngeal carcinoma, hepatocellular carcinoma, and neurodegenerative diseases, but not in spinal tuberculosis [11–13]. MR-PWI which directly affects the quantitative analysis and judgment on the Time-signal intensity curve (TIC) may help to identify parameters closely associated with the progress of spinal tuberculosis.

In the study, a spinal tuberculosis rabbit model was established, and the diffusion-weighted imaging and blood flow status changes in lumbar spine were observed and analyzed by PWI. These results may provide a reliable hemodynamic basis for the diagnosis of early spinal tuberculosis.

## Methods

### Bacteria, animals and groups

This study has been approval by Ethics Committees of the of the Second Affiliated Hospital of XinJiang Medical University (approval number: 2015022599). All methods were carried out in accordance with relevant guidelines and regulations.

The *Mycobacterium tuberculosis* (MTB) H37Rv (Shanghai Institute of Biological Products Co. Ltd, China) was cultured in a modified Sauton medium (PH = 7.2) for 2 to 3 weeks, then 5 mg/ml suspension was prepared.

Forty adult New Zealand white rabbits (male or female, aged 24 weeks, weighted 3.0–3.5 kg), were reared in separate cages for two weeks. The rabbits were randomly divided into two groups: the experiment group (n = 30) received lumbar facet injection of 0.2 ml tuberculosis suspension (5.0 mg/ml) and the control group (n = 10) received lumbar facet injection of 0.2 ml of normal saline. The X-rays of the rabbit spine were taken before infection to eliminate spinal anomaly.

### MRI technique

MRI scans were performed at 4, 6, and 8 weeks after infection. First, rabbits were anesthetized by injecting 2 mL 2% pentobarbital sodium along the ear edge of the rabbit. After anesthesia, rabbits were fixed in the prone position and scanned from L1 to S5 by dynamic enhanced magnetic resonance imaging using a Philips Gyroscan 1.5-T MRI scanner (Philips, Ltd, Best, the Netherlands). Before PWI studies, conventional MR images, including T1-weighted, T2-weighted, and fat suppressed T2WI (T2WI-SPIR) were acquired with a spin-echo pulse sequence. The T1WI sequence adopts a fast spin echo sequence: TR = 400 ms, TE = 6.2 ms, FOV = 261 mm, number of signal acquisitions (NSA) = 3 times, matrix = 256 × 256, slice thickness = 2.0 mm, and slice spacing = 1 mm. The T2WI sequence also uses a fast spin echo sequence: TR = 1171 ms, TE = 110 ms, FOV = 261 mm, NSA = 3 times, matrix = 512 × 512, slice thickness = 2.0 mm, and slice spacing = 1 mm. T2WI-SPIR: TR = 3000 ms, TE = 90 ms, FOV = 272 mm, NSA = 4 times, matrix = 248 × 240, slice thickness = 2.0 mm, slice spacing = 1 mm.

PWI was acquired from the maximal viewing position showing the destruction of the vertebral body in the sagittal view using a fast low-angle excitation on the high-pressure automatic Injector (Spectris MR Injector System, Medrad, USA). The parameter settings of PWI are as follows: TR = 6.8 ms, TE = 3.4 ms, FOV = 170 mm, slice thickness = 2 mm and scan time = 3 min. A paramagnetic contrast agent of 0.2–0.6 mmol/kg Gadopentetic acid (Gd-DTPA) [14] was injected through auricular vein with a 22-gauge cannula at a rate of 1–2 ml/s followed by 10 mL saline.

### PWI processing and calculation

At 4, 6, and 8 weeks after the infection, comparative measurement of the original data on L6 in the two groups was performed by two senior imaging physicians with blinding method. The central coronal surface of the bony side edge of the L6 vertebral in the surgical implantation area was defined as the region of interest (ROI), and the TIC was automatically generated by the computer. According to the parameters in TIC, the semi-quantitative parameters of the bone destruction area in the two groups were compared, including early enhancement parameters: first-pass enhancement rate (Efirst), first-pass enhancement velocity (Vfirst), early enhancement rate (Ee), early enhancement velocity (Ve), peak parameters: peak height (PH), time to peak (TTP), maximum slope of increase (MSI), maximum signal enhancement rate (Emax), signal enhancement rate (SER), and outflow parameter of washout.

### Statistical analysis

Data was expressed as mean ± standard deviation (SD). Statistical analysis was carried out with the SPSS20.0 software (IBM, USA). Normality test and variance analysis of data was performed. One-way ANOVA was performed when the variances were equal, the LSD t-test was performed for the comparison of means between the two groups, and the SNK-q test was used for the comparison of means among different time points in the experiment group. *P* < 0.05 was statistically significant. The receiver operating characteristic (ROC) curve analysis was applied to judge the accuracy of MR-perfusion parameters in the early diagnosis of spinal tuberculosis.

## Results

### Survival rate of rabbits

A total of forty adult New Zealand white rabbits were used in this experiment, including 30 in the model group and 40 in the control group. After the model was established, 4 rabbits in the control group died. One of the rabbits died of incision infection, swelling and pus; two rabbits died after operation due to poor eating and listlessness; another rabbit died 4 weeks after the operation by PCR. One rabbit in the control group died during the examination after intravenous injection of anesthesia. Finally, 26 rabbits were included in the model group and 9 rabbits were included in the control group.

### MRI perfusion images of the experiment group at 4, 6, and 8 weeks after infection

**Fig. 1:**
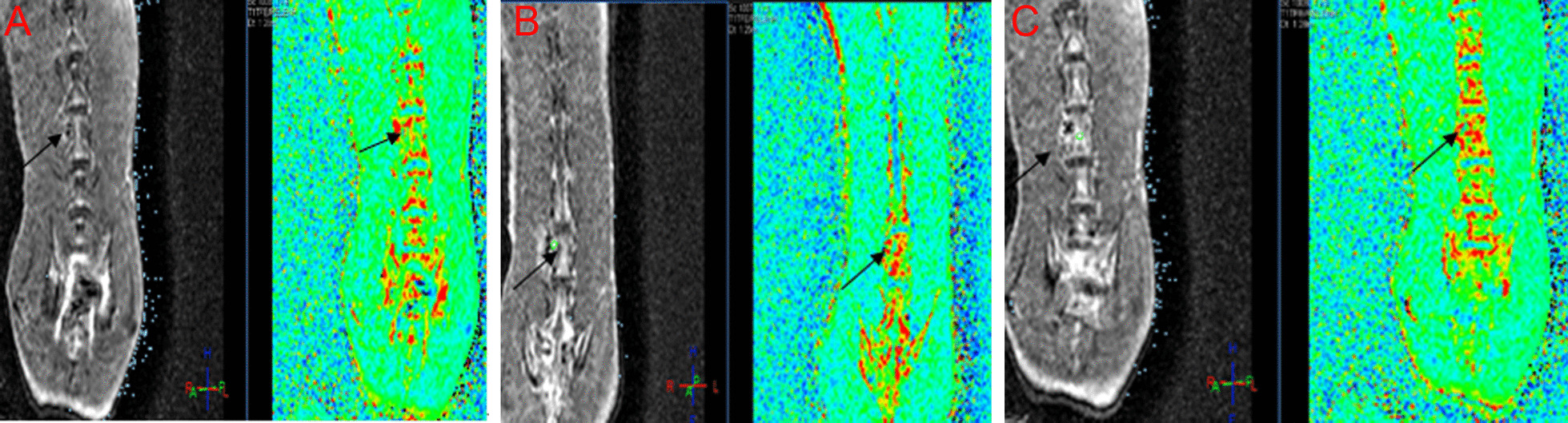
The characteristics of the dynamic changes of blood flow perfusion of microvessels inside the vertebra of spinal tuberculosis disease at 4 (**A**), 6 (**B**) and 8 (**C**) after weeks of infection in the experiment group. The left is the scan image of the region of interest (ROI), and the right is the pseudo-color image

The characteristics of the dynamic changes of blood flow perfusion of microvessels inside the vertebra of spinal tuberculosis over time were shown in Fig. 1A–C. Four weeks after infection, the blood perfusion map showed scattered spots (indicated by the arrows) in the tuberculosis implantation area, and the high blood perfusion signal was mostly concentrated around the ROI area (red spots). Six weeks after infection, the blood perfusion area was enlarged compared with 4 weeks after infection, and the high blood perfusion signal was concentrated inside the vertebra, showing a small patch of high perfusion, the perfusion amplitude was larger than that at 4 weeks postoperatively. The results showed that the blood perfusion area in the ROI area was increased significantly 8 weeks after the infection. There was a large area of high perfusion (represented in red), and there was also a high signal of blood perfusion inside the adjacent vertebral body.

### The TIC parameters

**Fig. 2:**
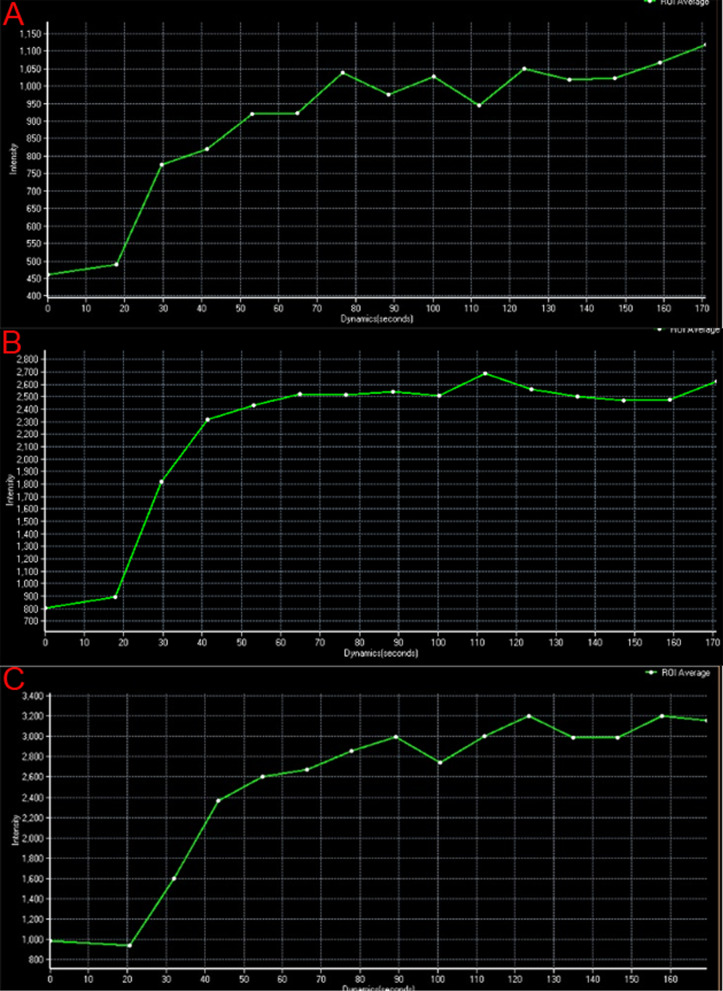
The time-signal intensity curve (TIC) of magnetic resonance (MR)- Perfusion weighted imaging (PWI) at 4 (**A**), 6 (**B**) and 8 (**C**) weeks after infection in the experiment group

At 4, 6, and 8 weeks after infection, the TICs were all showed a fast initial enhancement, followed by a plateau phase. The maximum signal intensity of the experiment group was 1050 (%/s), 2700 (%/s) and 3200 (%/s) at 4, 6 and 8 weeks after infection, respectively (Fig. 2). At the same time point, the signal intensity value of the MRI perfusion curve in the experiment group at 8 weeks postoperatively increased the most, and with the extension of time, the signal intensity gradually increased within the same time point.

### The early enhancement parameters were mainly increased after infection

The Efirst, Vfirst, Ee and Ve of the experiment group were statistically increased from 4 to 8 weeks after infection (all *P* < 0.05), and these parameters were also increased over time in the control group except for Ve. The Ve in the control group was decreased over time after infection, and the difference between 4 and 8 weeks after the infection was significant (*P* < 0.05). The four parameters in the experiment group at each time point were higher than those in the control group (all *P* < 0.05), except that Ee and Ve of the two groups at 4 weeks after the infection was similar (*P* > 0.05) (Table 1).

**Table 1:** The comparison of parameters between the experiment group and the control group after 4, 6 and 8 weeks after operation ($\overline{x} \pm s$, %)

Parameter	Group	4 weeks	6 weeks	8 weeks	F	*P*
Efirst	Experiment group (n = 26)	0.94 ± 0.37	1.51 ± 0.29	1.95 ± 0.43	58.060	0.000
	Control group (n = 9)	0.34 ± 0.26	0.55 ± 0.23	0.93 ± 0.14	58.138	0.000
	t	7.311	14.213	12.556	139.699	0.000
	P	0.000	0.000	0.000		
Vfirst	Experiment group (n = 26)	0.96 ± 0.27	1.26 ± 0.15	1.95 ± 0.23	155.370	0.000
	Control group (n = 9)	0.48 ± 0.15	0.57 ± 0.12	0.85 ± 0.19	45.589	0.000
	t	8.685	19.304	20.456	182.234	0.000
	P	0.000	0.000	0.000		
Ee	Experiment group (n = 26)	0.75 ± 0.15	0.97 ± 0.13	1.32 ± 0.32	52.591	0.000
	Control group (n = 9)	0.73 ± 0.16	0.75 ± 0.15	0.95 ± 0.11	22.037	0.000
	t	0.490	5.941	5.988	74.540	0.000
	P	0.626	0.000	0.000		
Ve	Experiment group (n = 26)	1.00 ± 0.22	1.21 ± 0.17	1.33 ± 0.20	20.786	0.000
	Control group (n = 9)	0.95 ± 0.12	0.90 ± 0.13	0.84 ± 0.12	5.832	0.004
	t	1.181	7.956	11.458	7.474	0.001
	P	0.243	0.000	0.000		
PH	Experiment group (n = 26)	941.00 ± 109.27	1448.67 ± 175.97	2191.47 ± 1002.39	33.977	0.000
	Control group (n = 9)	749.00 ± 91.01	1042.67 ± 128.89	1148.93 ± 105.62	107.130	0.000
	t	7.394	10.195	5.665	56.998	0.000
	P	0.000	0.000	0.000		
TTP	Experiment group (n = 26)	31.06 ± 1.03	33.53 ± 1.45	38.61 ± 4.93	95.791	0.000
	Control group (n = 9)	33.42 ± 1.45	38.92 ± 1.70	42.71 ± 4.68	79.754	0.000
	t	7.288	8.910	0.881	135.660	0.000
	P	0.000	0.000	0.382		
MSI	Experiment group (n = 26)	1.07 ± 0.23	1.49 ± 0.29	1.92 ± 0.47	45.506	0.000
	Control group (n = 9)	0.83 ± 0.29	1.08 ± 0.25	1.19 ± 0.15	17.609	0.000
	t	3.608	5.903	8.124	77.663	0.000
	P	0.001	0.000	0.000		
Emax	Experiment group (n = 26)	1.18 ± 0.28	1.69 ± 0.56	2.84 ± 0.80	63.471	0.000
	Control group (n = 9)	0.86 ± 0.19	1.44 ± 0.49	1.61 ± 0.42	31.296	0.000
	t	5.255	1.858	7.533	98.108	0.000
	P	0.000	0.068	0.000		
SER	Experiment group (n = 26)	1.02 ± 0.29	1.52 ± 0.39	1.94 ± 0.50	39.373	0.000
	Control group (n = 9)	0.87 ± 0.24	1.04 ± 0.25	1.20 ± 0.25	14.192	0.000
	t	2.159	5.606	7.228	57.121	0.000
	P	0.035	0.000	0.000		
washout	Experiment group (n = 26)	13.00 ± 4.87	16.07 ± 4.07	31.82 ± 4.50	151.756	0.000
	Control group (n = 9)	19.70 ± 5.04	18.19 ± 4.20	23.23 ± 6.56	7.012	0.002
	t	5.234	1.985	5.916	118.766	0.000
	P	0.000	0.052	0.000		

*Efirst* First-pass enhancement rate, *Vfirst* First-pass enhancement velocity, *Ee* Early enhancement rate, *Ve* Early enhancement velocity, *PH* peak height, *TTP* time to peak, *MSI* maximum slope of increase, *Emax* Maximum signal enhancement rate, *SER* Signal enhancement ratio

### The peak parameters were mainly increased after infection

The PH, MSI, SER and Emax in both two groups were significantly increased with time after infection (all *P* < 0.05), and those in the experiment group were higher than those in the control group at each time point (*P* < 0.05). It is worth remarkable that TTP was increased in two groups with time, but that in the experiment group was lower than that in the control group at each time point (all *P* < 0.05) (Table 1).

### The outflow parameter was increased in the experiment group than that in the control group

The washout rate of both two groups were significantly increased from 4 to 8 weeks after infection (*P* < 0.05). The washout rates of the experiment group were lower than those of the control group at 4 and 6 weeks after infection, while it was higher than that of the control group at 8 weeks after infection (*P* < 0.05) (Table 1).

### ROC curve analysis of MR-PWI parameters in diagnosing spinal tuberculosis

**Fig. 3:**
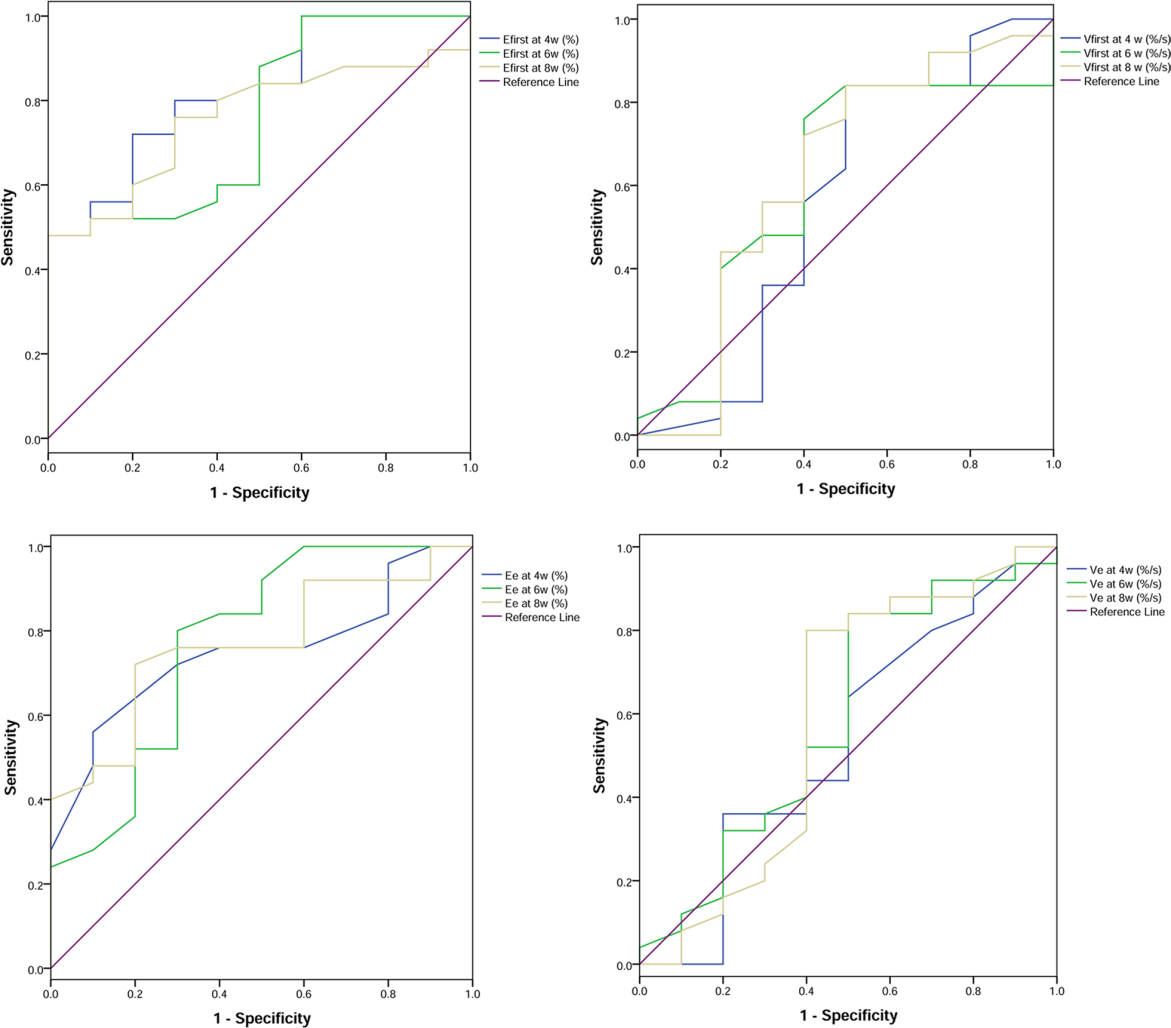
The receiver operating characteristic (ROC) curve analysis of early enhancement parameters. Efirst, First-pass enhancement rate; Vfirst, First-pass enhancement velocity; Ee, Early enhancement rate; Ve, Early enhancement velocity

At 4, 6, and 8 weeks after infection, the ROC curve was used to identify the best MR-PWI parameters for the diagnosis of spinal tuberculosis in the early stage. As shown in Table 2 and Fig. 3, of the four early enhancement parameters, the diagnosis values of Efirst and Ee were significant (*P* < 0.05). The AUC of Efirst at 4 weeks, 6 weeks and 8 weeks were 0.82 (95%*CI*: 0.68–0.97, *P* < 0.05), 0.76 (95%*CI*: 0.58–0.93, *P* < 0.05) and 0.76 (95%*CI*: 0.60–0.92, *P* < 0.05), respectively. The sensitivity and specificity of Efirst at 4 weeks were 72% and 80%, respectively. The sensitivity was reduced to 48% at 6 weeks and 8 weeks, while the specificity increased to 100%. The AUC of Ee at 4 weeks, 6 weeks and 8 weeks was ranging from 0.75 to 0.77. The sensitivities of Ee at 4 weeks, 6 weeks and 8 weeks were 56%, 80% and 72%, respectively. The specificities of Ee at 4 weeks, 6 weeks and 8 weeks were 90%, 70% and 80%, respectively.

**Table 2:** Receiver operating characteristic (ROC) analysis of early enhancement parameters in diagnosing early-phase spinal tuberculosis

Variables		AUC	95%CI	Sensitivity (%)	Specificity (%)	Cut-off value	Y-index	*P*
Efirst	4 weeks	0.82	(0.68, 0.97)	72.00	80.00	≥ 0.73	0.52	0.003
	6 weeks	0.76	(0.58, 0.93)	48.00	100.00	≥ 1.54	0.48	0.019
	8 weeks	0.76	(0.60, 0.92)	48.00	100.00	≥ 2.02	0.48	0.019
Vfirst	4 weeks	0.56	(0.31, 0.81)	84.00	50.00	≥ 0.64	0.34	0.596
	6 weeks	0.61	(0.38, 0.83)	76.00	60.00	≥ 1.15	0.36	0.333
	8 weeks	0.62	(0.39, 0.86)	84.00	50.00	≥ 1.38	0.34	0.258
Ee	4 weeks	0.75	(0.59, 0.91)	56.00	90.00	≥ 0.77	0.46	0.022
	6 weeks	0.77	(0.58, 0.96)	80.00	70.00	≥ 0.88	0.50	0.013
	8 weeks	0.77	(0.61, 0.93)	72.00	80.00	≥ 1.15	0.52	0.014
Ve	4 weeks	0.53	(0.30, 0.76)	36.00	80.00	≥ 1.05	0.16	0.784
	6 weeks	0.59	(0.36, 0.82)	84.00	50.00	≥ 1.01	0.34	0.411
	8 weeks	0.59	(0.34, 0.84)	80.00	60.00	≥ 1.20	0.40	0.411

*AUC* area under ROC curve, *95%CI* 95% Confidence interval, *Efirst* First-pass enhancement rate, *Vfirst* First-pass enhancement velocity, *Ee* Early enhancement rate, *Ve* Early enhancement velocity

**Fig. 4:**
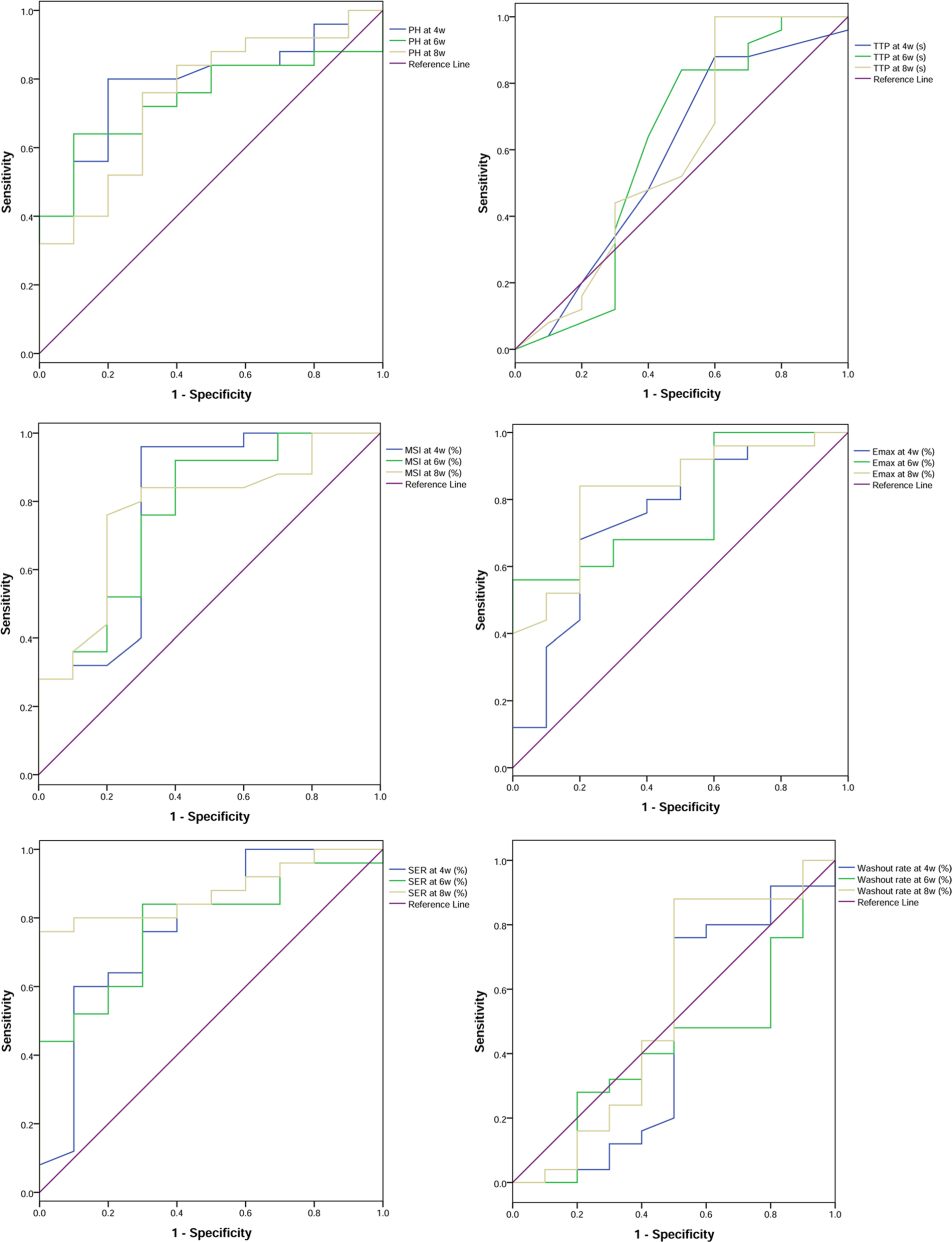
The receiver operating characteristic (ROC) curve analysis of peak parameters and washout. *PH* peak height, *TTP* time to peak, *MSI* maximum slope of increase; *Emax* Maximum signal enhancement rate, *SER* Signal enhancement ratio

Among the 5 peak parameters, the diagnosis values of PH, MSI, Emax and SER were significant (P < 0.05). The AUCs of all these four parameters were larger than 0.7 (Table 3 and Fig. 4). Besides, the sensitivity and specificity of these parameters were relatively high with a range between 56 and 100%. However, the washout was not significant in diagnosing spinal tuberculosis (*P* > 0.05) (Table 3 and Fig. 4).

**Table 3:** Receiver operating characteristic (ROC) analysis of peak parameters and Washout in diagnosing early-phase spinal tuberculosis

Variables		AUC	95%CI	Sensitivity (%)	Specificity (%)	Cut-off value	Y-index	*P*
PH	4 weeks	0.78	(0.62, 0.94)	80.00	80.00	≥ 865.00	0.60	0.010
	6 weeks	0.74	(0.58, 0.91)	64.00	90.00	≥ 1402.00	0.54	0.026
	8 weeks	0.75	(0.57, 0.93)	76.00	70.00	≥ 1476.50	0.46	0.024
TTP	4 weeks	0.58	(0.35, 0.81)	88.00	40.00	≤ 32.50	0.28	0.454
	6 weeks	0.60	(0.35, 0.85)	84.00	50.00	≤ 36.00	0.34	0.342
	8 weeks	0.59	(0.35, 0.84)	100.00	40.00	≤ 44.50	0.40	0.391
MSI	4 weeks	0.78	(0.58, 0.98)	96.00	70.00	≥ 0.90	0.66	0.010
	6 weeks	0.77	(0.58, 0.95)	92.00	60.00	≥ 1.21	0.52	0.014
	8 weeks	0.77	(0.60, 0.95)	76.00	80.00	≥ 1.72	0.56	0.013
Emax	4 weeks	0.75	(0.56, 0.94)	68.00	80.00	≥ 1.07	0.48	0.021
	6 weeks	0.78	(0.62, 0.94)	56.00	100.00	≥ 1.66	0.56	0.012
	8 weeks	0.83	(0.68, 0.97)	84.00	80.00	≥ 2.28	0.64	0.003
SER	4 weeks	0.78	(0.60, 0.97)	60.00	90.00	≥ 1.01	0.50	0.010
	6 weeks	0.78	(0.62, 0.94)	84.00	70.00	≥ 1.22	0.54	0.011
	8 weeks	0.88	(0.76, 0.99)	76.00	100.00	≥ 1.78	0.76	0.001
Washout	4 weeks	0.46	(0.20, 0.71)	76.00	50.00	≥ 11.49	0.26	0.701
	6 weeks	0.42	(0.20, 0.64)	100.00	10.00	≥ 12.84	0.10	0.465
	8 weeks	0.54	(0.29, 0.79)	88.00	50.00	≥ 25.25	0.38	0.715

*AUC* area under ROC curve, *95%CI* 95% Confidence interval, *PH* peak height, *TTP* time to peak, *MSI* maximum slope of increase, *Emax* Maximum signal enhancement rate, *SER* Signal enhancement ratio

## Discussion

Early rapid and accurate diagnose of spinal tuberculosis still remains a challenge currently [15, 16]. In this study, MR-PWI was performed to evaluate the various parameters of hemodynamics in early diagnosis of spinal tuberculosis. The results of MR-PWI showed that the MRI perfusion parameters in the experiment group were mostly increase with time after the operation, including Efirst, MSI, Ee, PH, Emax, SER and washout. After the ROC curve analysis, Efirst, Ee, PH, MSI, Emax and SER showed significant values for early diagnosing spinal tuberculosis with relative high sensitivity and specificity.

The TIC curve analysis displayed that at 4, 6, and 8 weeks after surgery, the early perfusion parameters of MRI mostly increase with time, especially those in the experiment group (all *P* < 0.05). This inferred that the rabbits of the experiment group were successfully infected by MTB H37Rv, and the early perfusion parameters were correlated with the blood supply of the pathological tissue. Efirst and Ee represent the signal enhancement degree in the diseased vertebra when the artery reaches its peak, and the degree of vascularization of the diseased vertebral body is the main factor of enhancement [17]. Verstraete et al. [18] believed that the first-pass slope which connected with blood and vessel distribution can provide quantitative information in tissue blood perfusion. The TIC of the ROI in the experiment group was mostly showed a fast initial enhancement, followed by a plateau phase, which is similar to the curve of benign lesions of most skeletal system in the literature [19]. MSI was reached the highest level 8 weeks after infection in the experiment group, this results indicated that the blood supply inside the diseased vertebral body was increased with time [20]. Emax is the saturation point at which perfusion and leakage reach a balance, and the latest Emax which was increased with time in the study could represent the volume of microvessels inside the lesion [21]. The previous literature reported that SER affected by blood vessels is appeared in the equilibrium period of dynamic enhancement, which mainly reflected the maximum ability of the tissue to accumulate contrast agent [22–24]. In this experiment, the SER of the diseased vertebra in the experiment group was gradually increased with time, and was highest at 8 weeks after infection. This suggested that the abundant blood supply inside the diseased vertebra at 8 weeks after infection. PH is not only related to the volume of capillaries and tiny veins inside the diseased tissue, but also related to the volume of the intercellular space outside the blood vessel [25, 26]. The level of PH in the experiment group at 8 weeks was highest compared with the other two time points, this indicated that the total amount of the internal microcirculation volume and the extravascular extracellular space of the diseased vertebral body was the highest. The TTP of the experiment group was significantly shorter than that in the control group at 4, 6, and 8 weeks after infection, the results is consistent with previous study that the higher the microvessel density, the shorter the TTP [27]. The outflow quantifying the degree of enhancement of the descending segment in the TIC curve is rarely mentioned in literature. When the capillary permeability is high, excretion is rapid, thus the excretion rate (washout) is a parameter that should be considered in clinical diagnosis [28, 29].The washout value of the experiment group is higher than that of the control group within 8 weeks after surgery, which indicated that the distribution of microvessels in the diseased vertebral body of it was more than that of the control group.

The cut-off values of perfusion parameters have not a uniform standard. For this reason, the ROC curve which is usually used for medical diagnostic test evaluation was conducted to identify candidate parameters for diagnosing and monitoring spinal tuberculosis. After the ROC curve analysis of various MR-PWI parameters, the results showed that Efirst, Ee, PH, MSI, Emax and SER might be candidate biomarkers for diagnosing spinal tuberculosis with relative high sensitivity and specificity, while the parameters of Vfirst, Ve, TTP and washout were not significant.

## Conclusion

In conclusion, the parameters including Efirst, Ee, PH, MSI, Emax and SER might be applied for early diagnosis of spinal tuberculosis. This analysis not only quantitatively determined the role of various MR-PWI parameters of in the early diagnosis of spinal tuberculosis, but also provided a basis for choosing appropriate perfusion parameters and determining the diagnostic threshold standard for early diagnosis of spinal tuberculosis in the future.

## Data Availability

The data that support the findings of this study are available on request from the corresponding author.

## Abbreviations

MR: Magnetic resonance
PWI: Perfusion weighted imaging
ROC: Receiver operating characteristic
Efirst: First-pass enhancement rate
Ee: Early enhancement rate
PH: Peak height
MSI: Maximum slope of increase
Emax: Maximum signal enhancement rate
SER: Signal enhancement rate
TIC: Time-signal intensity curve
ROI: Region of interest
Vfirst: First-pass enhancement velocity
Ve: Early enhancement velocity
TTP: Time to peak
SD: Standard deviation
